# Supplementary material for: Development of a knowledge translation platform for ataxia: Impact on readers and volunteer contributors
Source: PLoS One. 2020 Sep 1;15(9):e0238512. doi: 10.1371/journal.pone.0238512 (PMC7462291; doi:10.1371/journal.pone.0238512)
Supplement: S1 File — (DOCX) [file pone.0238512.s001.docx]

**S1. Evaluating the Impact of a Knowledge Translation Platform for Spinocerebellar Ataxias on its Volunteers – Survey Questions**

All surveys were administered through the LimeSurvey platform.

**Contributor Survey Questions**

Please select which of the following best describes your current position:

- Graduate Student
- Postdoctoral Fellow
- Primary Investigator
- Other (Recent Graduate, Laboratory technician, etc.)
- I prefer not to disclose

How many SCAsource articles or snapshots have you contributed to as a writer or editor during your time as a volunteer?

- 1
- 2-3
- 4-5
- 6+

Please respond to the next 4 statements using the following scale: 1 – Strongly Disagree, 2 – Disagree, 3 – Neutral, 4 – Agree, 5 – Strongly Agree.

- Volunteering for SCAsource has improved my writing and/or editing skills
- Volunteering for SCAsource has given me more confidence in communicating scientific findings to a lay audience
- Volunteering for SCAsource has increased the amount of time I dedicate to knowledge translation over the course of a year
- Volunteering for SCAsource has been beneficial to my development as a scientist
- Volunteering for SCAsource has improved my time management skills
- Volunteering for SCAsource has enhanced my understanding of SCA and ataxia research literature
- Volunteering for SCAsource has helped me give back to the SCA and ataxia patient community

Do you visit the SCAsource Website (scasource.net)?

- No
- Yes
- Yes, but only to check if my article(s) have been published

If you visit scasource.net to read and check for articles, how often do you visit on average?

- More than once a week
- Once a week
- Once a month
- Once every few months
- Once a year
- Less than once a year
- Not applicable

How has volunteering for SCAsource impacted your skills at communicating scientific finding to the general public? [Short answer]

What aspects of SCAsource have been done well? [Short answer]

What aspects of SCAsource could be improved? [Short answer]

Do you have any further comments on SCAsource? [short answer]

**Closing Statement**

Thank you for taking this survey. Your answers are a valuable part of this research.

**Reader Survey Questions**

How many SCAsource articles have you read in the last year?

- 1-2
- 3-4
- 5-6
- 7+

How often do you search for information on ataxia or ataxia research online?

- More than once a week
- Once a week
- Once a month
- Once every few months
- Once a year
- Less than once a year

What are your main source(s) for finding information about ataxia and ataxia research online?

- SCAsource website
- SCAsource email list
- Google / Bing / Yahoo / Other Search Engine
- The National Ataxia Foundation
- Facebook
- Twitter
- Shared by friends or family
- Other (Please describe)

What is your main method(s) of finding out about new SCAsource article? (select all that apply)

- SCAsource website
- SCAsource email list
- Google / Bing / Yahoo / Other Search Engine
- The National Ataxia Foundation
- Facebook
- Twitter
- Shared by friends or family
- Other (Please describe)

If you visit scasource.net directly to read and check for articles and snapshots, how often do you visit on average?

- Multiple time a week
- Every week
- Every two weeks
- Once a month
- Once every two or three months
- Once a year

Please respond to the next 4 statements using the following scale: 1 – Strongly Disagree, 2 – Disagree, 3 – Neutral, 4 – Agree, 5 – Strongly Agree.

- Reading SCAsource content has helped me better understand what research is being conducted on ataxia
- I have learned more about ataxia by reading SCAsource
- After reading SCAsource content, I feel more connected to research that is being conducted on ataxia
- I trust SCAsource to have unbiased writing of ataxia research news
- Reading SCAsource content has made me more interested in getting involved in ataxia research and/or clinical trials

Please rate how helpful you found the following SCAsource content when learning more about ataxia and ataxia research using the following scale: 1 – Extremely helpful, 2 – very helpful, 3 – Moderately helpful, 4 – Slightly helpful, 5 – Not at all helpful.

- SCAsource Summaries
- SCAsource Snapshots
- Glossary
- What is Ataxia? Information Page

What do you like most about SCAsource content? [short answer]

What about SCAsource content could be improved? [short answer]

Do you have any suggestions for future topics for SCAsource articles or snapshots? [short answer]

Do you have any further comments on SCAsource? [short answer]
